# Absolute configuration of (1*S*,3*R*,8*R*)-10-bromo­methyl-2,2-di­chloro-3,7,7-tri­methyl­tri­cyclo­[6.4.0.0^1,3^]dodec-9-ene

**DOI:** 10.1107/S1600536813028183

**Published:** 2013-10-23

**Authors:** Abdoullah Bimoussa, Aziz Auhmani, My Youssef Ait Itto, Jean-Claude Daran, Abdelwahed Auhmani

**Affiliations:** a Laboratoire de Physico-Chimie Moléculaire et Synthése Organique, Département de Chimie Faculté des Sciences, Semlalia BP 2390, Marrakech 40001, Morocco; b Laboratoire de Chimie de Coordination, 205 route de Narbonne, 31077 Toulouse Cedex 04, France

## Abstract

The absolute configuration of the title compound, C_16_H_23_BrCl_2_, has been deduced from the chemical pathway and fully confirmed by refinement of the Flack and Hooft parameters. The six-membered ring adopts a half-chair conformation, whereas the seven-membered ring is a twisted chair. The mol­ecular packing within the crystal is stabilized only by van der Waals inter­actions.

## Related literature

For the synthesis of the title compound, see: El Jamili *et al.* (2002 ▶). For further synthetic details, see: Qu *et al.* (2009 ▶). For biological properties of cyclo­propane-containing products, see: Ajay Kumar *et al.* (2012 ▶); Sow *et al.* (2007 ▶); Symon *et al.* (2005 ▶). For related structures, see: Benharref *et al.* (2010 ▶); Gassman & Gorman (1990 ▶); Lassaba *et al.* (1997 ▶). For conformations of rings, see: Cremer & Pople (1975 ▶); Boessenkool & Boyens (1980 ▶); For absolute structure, see: Flack (1983 ▶); Flack & Bernardinelli (2000 ▶); Spek (2009 ▶).
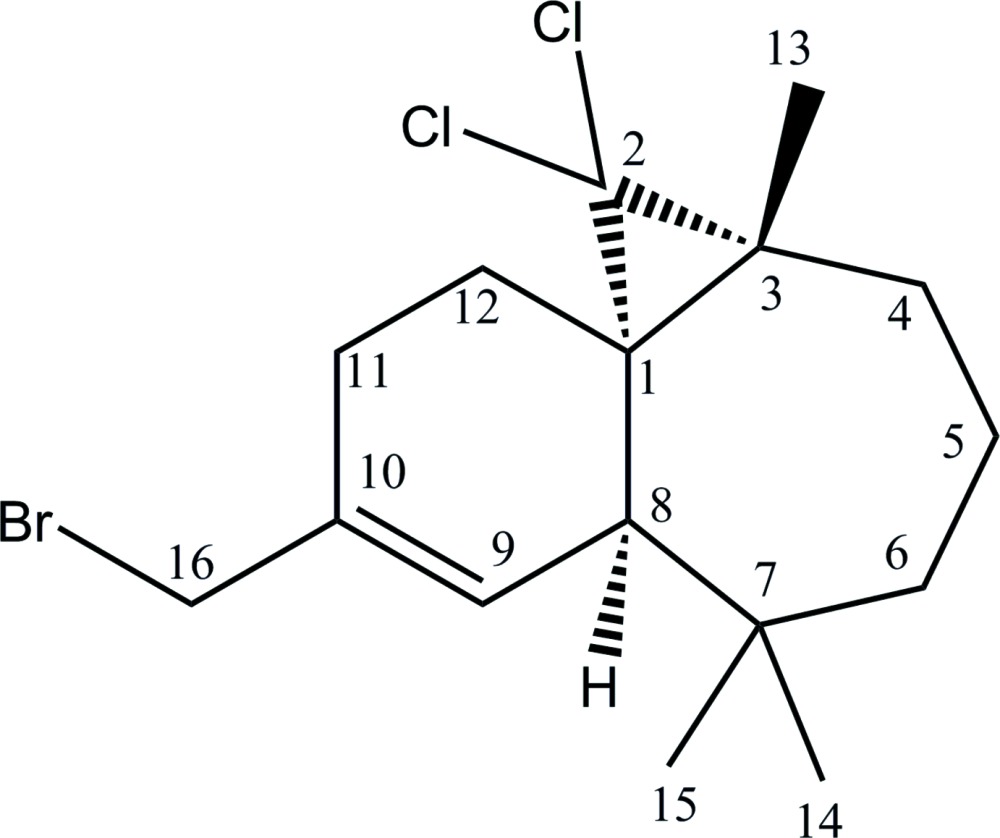


## Experimental

### 

#### Crystal data

C_16_H_23_BrCl_2_
*M* *_r_* = 366.15
Orthorhombic, 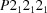

*a* = 9.1000 (2) Å
*b* = 12.5490 (4) Å
*c* = 14.4070 (5) Å
*V* = 1645.22 (9) Å^3^
*Z* = 4
Cu *K*α radiation
μ = 6.26 mm^−1^
*T* = 173 K
0.45 × 0.25 × 0.10 mm

#### Data collection

Agilent Xcalibur Gemini ultra diffractometer
Absorption correction: multi-scan (*CrysAlis PRO*; Agilent, 2012 ▶) *T*_min_ = 0.397, *T*_max_ = 1.000
9248 measured reflections
3127 independent reflections
3012 reflections with *I* > 2σ(*I*)
*R*_int_ = 0.038

#### Refinement

*R*[*F* ^2^ > 2σ(*F* ^2^)] = 0.027
*wR*(*F* ^2^) = 0.070
*S* = 1.04
3127 reflections
176 parameters
H-atom parameters constrained
Δρ_max_ = 0.41 e Å^−3^
Δρ_min_ = −0.46 e Å^−3^
Absolute structure: Flack (1983 ▶), 1307 Friedel pairs
Absolute structure parameter: −0.015 (17)

### 

Data collection: *CrysAlis PRO* (Agilent, 2012 ▶); cell refinement: *CrysAlis PRO*; data reduction: *CrysAlis PRO*; program(s) used to solve structure: *SIR97* (Altomare *et al.*, 1999 ▶); program(s) used to refine structure: *SHELXL97* (Sheldrick, 2008 ▶); molecular graphics: *ORTEP-3 for Windows* (Farrugia, 2012 ▶); software used to prepare material for publication: *SHELXL97*.

## Supplementary Material

Crystal structure: contains datablock(s) I, New_Global_Publ_Block. DOI: 10.1107/S1600536813028183/yk2099sup1.cif

Structure factors: contains datablock(s) I. DOI: 10.1107/S1600536813028183/yk2099Isup2.hkl

Supplementary material file. DOI: 10.1107/S1600536813028183/yk2099Isup3.cml

Additional supplementary materials:  crystallographic information; 3D view; checkCIF report

## Figures and Tables

**Fig. 1.**
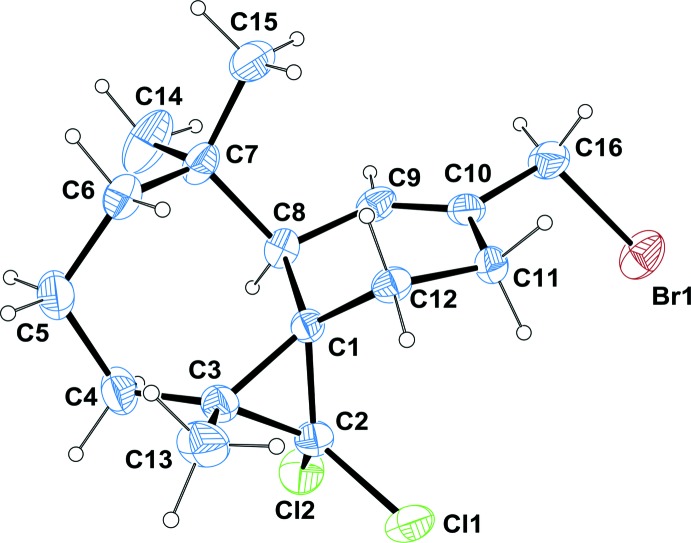
The asymmetric unit of (I) with the atom-labelling scheme. Displacement ellipsoids are drawn at the 50% probability level. H atoms are represented as small spheres of arbitrary radii.

## supplementary crystallographic information

### 1. Comment

Cyclopropane-containing natural products attract great interest because of their biological properties ranging from insecticidal (Sow *et al.*, 2007) and antimicrobial (Ajay Kumar *et al.*, 2012) to antitumoral activities (Symon *et al.*, 2005). They are also valuable intermediates and are frequently used as versatile building blocks in organic synthesis (Qu *et al.*, 2009).

In our ongoing studies on the synthesis of new chiral sesquiterpenic cyclopropanes, we carried out the reaction of (1*S*,3*R*,8*R*)-2,2-dichloro-3,7,7-10-tetramethyltricyclo[6.4.0.0^1,3^]dodec-9-ene (El Jamili *et al.*, 2002) with *N*-bromosuccinimide (NBS) and obtained the title compound in poor yield. Its gross structure was confirmed by spectroscopic data, and its stereochemistry was fully established as (1*S*,3*R*,8*R*) to prove no racemization during the reaction process.

A view of the molecule is represented in Fig. 1. As observed in related compounds (Gassman & Gorman, 1990; Lassaba *et al.*, 1997; Benharref *et al.*, 2010), each molecule is built up from two fused six-and seven-membered rings. The six-membered ring has roughly half-chair conformation with the puckering parameters: Q = 0.493 (3) Å, spherical polar angle θ= 131.2 (3)° and φ= 147.2 (4)° (Cremer & Pople, 1975), whereas the seven-membered ring displays a twisted chair conformation with a total puckering amplitude of 1.148 (3) Å. (Boessenkool & Boyens, 1980).

The absolute configuration (1*S*,3*R*,8*R*) deduced from the chemical pathway is confirmed by the refinement of the Flack's parameter, -0.015 (17), (Flack, 1983; Flack & Bernardinelli, 2000) and by the refinement of the Hooft's parameter, -0.021 (11) (Spek, 2009).

The packing of the molecules within the crystal is only stabilized by van der Waals interactions.

### 2. Experimental

To a cooled (0°C) solution of (1*S*,3*R*,8*R*)-2,2-dichloro-\3,7,7-10-tetramethyltricyclo[6.4.0.0^1,3^]dodec-9-ene (4,6 mmol) in 50 ml of a solvent mixture THF/H_2_O (4/1, *v*/*v*), NBS (9,16 mmol) was added in small portions, then mixture was kept under stirring at 0°C for two hours. After completion of the reaction, 15% sodium hydrogenocarbonate solution was added and the reaction mixture was taken up in ether, dried over anhydrous sodium sulfate, and concentrated. The crude product was purified by chromatography on silica gel (230–400 mesh) with hexane as eluent to give the title compound in 9% yield.

### 3. Refinement

All H atoms attached to C atoms were fixed geometrically and treated as riding with C—H = 0.99 Å (methylene), 0.98 Å (methyl) and 1.0 Å (methine) with *U*_iso_(H) = 1.2*U*_eq_(CH and CH_2_) or *U*_iso_(H) = 1.5*U*_eq_(CH_3_).

### Crystal data

**Table:**

C_16_H_23_BrCl_2_	*F*(000) = 752
*M**_r_* = 366.15	*D*_x_ = 1.478 Mg m^−^^3^
Orthorhombic, *P*2_1_2_1_2_1_	Cu *K*α radiation, λ = 1.54184 Å
Hall symbol: P 2ac 2ab	Cell parameters from 5474 reflections
*a* = 9.1000 (2) Å	θ = 3.1–70.8°
*b* = 12.5490 (4) Å	µ = 6.26 mm^−^^1^
*c* = 14.4070 (5) Å	*T* = 173 K
*V* = 1645.22 (9) Å^3^	Box, colourless
*Z* = 4	0.45 × 0.25 × 0.10 mm

### Data collection

**Table:**

Agilent Xcalibur Gemini ultra diffractometer	3127 independent reflections
Radiation source: Enhance Ultra (Cu) X-ray Source	3012 reflections with *I* > 2σ(*I*)
Mirror monochromator	*R*_int_ = 0.038
Detector resolution: 16.1978 pixels mm^-1^	θ_max_ = 70.9°, θ_min_ = 4.7°
ω scans	*h* = −11→11
Absorption correction: multi-scan (*CrysAlis PRO*; Agilent, 2012)	*k* = −15→14
*T*_min_ = 0.397, *T*_max_ = 1.000	*l* = −17→17
9248 measured reflections	

### Refinement

**Table:**

Refinement on *F*^2^	Hydrogen site location: inferred from neighbouring sites
Least-squares matrix: full	H-atom parameters constrained
*R*[*F*^2^ > 2σ(*F*^2^)] = 0.027	*w* = 1/[σ^2^(*F*_o_^2^) + (0.0372*P*)^2^ + 0.318*P*] where *P* = (*F*_o_^2^ + 2*F*_c_^2^)/3
*wR*(*F*^2^) = 0.070	(Δ/σ)_max_ < 0.001
*S* = 1.04	Δρ_max_ = 0.41 e Å^−^^3^
3127 reflections	Δρ_min_ = −0.46 e Å^−^^3^
176 parameters	Extinction correction: *SHELXL97* (Sheldrick, 2008), Fc^*^=kFc[1+0.001xFc^2^λ^3^/sin(2θ)]^-1/4^
0 restraints	Extinction coefficient: 0.0037 (2)
Primary atom site location: structure-invariant direct methods	Absolute structure: Flack (1983), 1307 Friedel pairs
Secondary atom site location: difference Fourier map	Absolute structure parameter: −0.015 (17)

### Special details

**Table:**

Geometry. All e.s.d.'s (except the e.s.d. in the dihedral angle between two l.s. planes) are estimated using the full covariance matrix. The cell e.s.d.'s are taken into account individually in the estimation of e.s.d.'s in distances, angles and torsion angles; correlations between e.s.d.'s in cell parameters are only used when they are defined by crystal symmetry. An approximate (isotropic) treatment of cell e.s.d.'s is used for estimating e.s.d.'s involving l.s. planes.
Refinement. Refinement of *F*^2^ against ALL reflections. The weighted *R*-factor *wR* and goodness of fit *S* are based on *F*^2^, conventional *R*-factors *R* are based on *F*, with *F* set to zero for negative *F*^2^. The threshold expression of *F*^2^ > 2σ(*F*^2^) is used only for calculating *R*-factors(gt) *etc*. and is not relevant to the choice of reflections for refinement. *R*-factors based on *F*^2^ are statistically about twice as large as those based on *F*, and *R*- factors based on ALL data will be even larger.

### Fractional atomic coordinates and isotropic or equivalent isotropic displacement parameters (Å^2^)

**Table:**

	*x*	*y*	*z*	*U*_iso_*/*U*_eq_	
C1	0.3341 (3)	0.3344 (2)	0.29478 (17)	0.0200 (5)	
C2	0.4125 (3)	0.2394 (2)	0.33667 (17)	0.0245 (5)	
C3	0.3927 (3)	0.3389 (2)	0.39476 (17)	0.0264 (5)	
C4	0.2786 (4)	0.3369 (2)	0.47129 (18)	0.0356 (6)	
H4A	0.3274	0.3208	0.5311	0.043*	
H4B	0.2068	0.2795	0.4586	0.043*	
C5	0.1975 (4)	0.4434 (2)	0.47910 (19)	0.0372 (6)	
H5A	0.2572	0.4925	0.5175	0.045*	
H5B	0.1035	0.4314	0.5120	0.045*	
C6	0.1646 (3)	0.4980 (2)	0.38583 (18)	0.0312 (6)	
H6A	0.1074	0.5634	0.3989	0.037*	
H6B	0.2594	0.5208	0.3586	0.037*	
C7	0.0814 (3)	0.4344 (2)	0.31168 (19)	0.0295 (6)	
C8	0.1674 (3)	0.3298 (2)	0.28177 (17)	0.0226 (5)	
H8	0.1312	0.2714	0.3231	0.027*	
C9	0.1316 (3)	0.2964 (2)	0.18373 (18)	0.0259 (5)	
H9	0.0338	0.2742	0.1709	0.031*	
C10	0.2264 (3)	0.2957 (2)	0.11464 (16)	0.0249 (5)	
C11	0.3846 (3)	0.3268 (2)	0.12612 (16)	0.0234 (5)	
H11A	0.4155	0.3702	0.0721	0.028*	
H11B	0.4460	0.2617	0.1273	0.028*	
C12	0.4109 (2)	0.39027 (19)	0.21497 (16)	0.0222 (5)	
H12A	0.5176	0.3952	0.2276	0.027*	
H12B	0.3717	0.4634	0.2079	0.027*	
C13	0.5259 (3)	0.4058 (2)	0.4181 (2)	0.0378 (7)	
H13A	0.4943	0.4778	0.4355	0.057*	
H13B	0.5907	0.4097	0.3639	0.057*	
H13C	0.5789	0.3733	0.4701	0.057*	
C14	−0.0692 (3)	0.4003 (3)	0.3467 (3)	0.0564 (10)	
H14A	−0.0577	0.3582	0.4036	0.085*	
H14B	−0.1181	0.3570	0.2993	0.085*	
H14C	−0.1286	0.4636	0.3599	0.085*	
C15	0.0576 (4)	0.5105 (2)	0.2286 (2)	0.0399 (7)	
H15A	0.0002	0.4742	0.1806	0.060*	
H15B	0.1530	0.5317	0.2031	0.060*	
H15C	0.0044	0.5741	0.2496	0.060*	
C16	0.1748 (3)	0.2646 (2)	0.01985 (19)	0.0339 (6)	
H16A	0.1982	0.3225	−0.0244	0.041*	
H16B	0.0667	0.2556	0.0208	0.041*	
Cl1	0.59023 (7)	0.20569 (6)	0.29648 (5)	0.03423 (16)	
Cl2	0.31861 (8)	0.12015 (5)	0.36321 (5)	0.03703 (17)	
Br1	0.26676 (3)	0.13107 (2)	−0.02346 (2)	0.04148 (11)	

### Atomic displacement parameters (Å^2^)

**Table:**

	*U*^11^	*U*^22^	*U*^33^	*U*^12^	*U*^13^	*U*^23^
C1	0.0227 (10)	0.0175 (11)	0.0199 (11)	0.0007 (9)	0.0004 (10)	−0.0003 (9)
C2	0.0243 (11)	0.0226 (12)	0.0266 (12)	0.0009 (10)	−0.0006 (10)	0.0019 (10)
C3	0.0342 (13)	0.0217 (12)	0.0232 (12)	0.0033 (11)	−0.0033 (10)	−0.0004 (9)
C4	0.0548 (16)	0.0300 (14)	0.0219 (11)	0.0021 (13)	0.0046 (14)	0.0007 (10)
C5	0.0516 (16)	0.0350 (15)	0.0249 (12)	0.0037 (13)	0.0037 (13)	−0.0042 (11)
C6	0.0341 (14)	0.0269 (14)	0.0327 (14)	0.0029 (12)	0.0033 (12)	−0.0063 (11)
C7	0.0223 (12)	0.0320 (14)	0.0342 (14)	0.0048 (11)	0.0042 (11)	−0.0070 (11)
C8	0.0197 (10)	0.0210 (13)	0.0272 (13)	−0.0046 (9)	0.0033 (10)	−0.0029 (9)
C9	0.0187 (11)	0.0244 (12)	0.0347 (14)	0.0017 (10)	−0.0040 (10)	−0.0052 (11)
C10	0.0284 (13)	0.0217 (11)	0.0247 (11)	0.0060 (11)	−0.0064 (10)	−0.0023 (9)
C11	0.0251 (12)	0.0246 (13)	0.0203 (11)	0.0018 (10)	0.0036 (9)	0.0019 (9)
C12	0.0178 (10)	0.0208 (12)	0.0280 (12)	−0.0003 (10)	0.0006 (9)	0.0014 (10)
C13	0.0402 (16)	0.0347 (16)	0.0383 (16)	0.0004 (13)	−0.0167 (13)	−0.0079 (12)
C14	0.0283 (15)	0.057 (2)	0.084 (3)	−0.0018 (15)	0.0225 (17)	−0.021 (2)
C15	0.0409 (15)	0.0380 (17)	0.0408 (16)	0.0156 (14)	−0.0075 (13)	−0.0083 (13)
C16	0.0414 (14)	0.0292 (14)	0.0313 (13)	0.0103 (12)	−0.0075 (13)	−0.0094 (12)
Cl1	0.0269 (3)	0.0328 (4)	0.0430 (4)	0.0090 (3)	−0.0042 (3)	−0.0005 (3)
Cl2	0.0493 (4)	0.0205 (3)	0.0413 (3)	−0.0027 (3)	0.0060 (3)	0.0046 (3)
Br1	0.03964 (16)	0.03661 (18)	0.04820 (18)	0.00297 (14)	−0.00203 (13)	−0.01953 (14)

### Geometric parameters (Å, º)

**Table:**

C1—C2	1.515 (3)	C8—H8	1.0000
C1—C12	1.518 (3)	C9—C10	1.317 (4)
C1—C8	1.529 (3)	C9—H9	0.9500
C1—C3	1.537 (3)	C10—C16	1.496 (3)
C2—C3	1.515 (3)	C10—C11	1.501 (3)
C2—Cl2	1.765 (3)	C11—C12	1.526 (3)
C2—Cl1	1.769 (3)	C11—H11A	0.9900
C3—C13	1.512 (4)	C11—H11B	0.9900
C3—C4	1.515 (4)	C12—H12A	0.9900
C4—C5	1.531 (4)	C12—H12B	0.9900
C4—H4A	0.9900	C13—H13A	0.9800
C4—H4B	0.9900	C13—H13B	0.9800
C5—C6	1.538 (4)	C13—H13C	0.9800
C5—H5A	0.9900	C14—H14A	0.9800
C5—H5B	0.9900	C14—H14B	0.9800
C6—C7	1.533 (4)	C14—H14C	0.9800
C6—H6A	0.9900	C15—H15A	0.9800
C6—H6B	0.9900	C15—H15B	0.9800
C7—C14	1.522 (4)	C15—H15C	0.9800
C7—C15	1.546 (4)	C16—Br1	1.974 (3)
C7—C8	1.589 (4)	C16—H16A	0.9900
C8—C9	1.509 (3)	C16—H16B	0.9900
			
C2—C1—C12	116.7 (2)	C9—C8—H8	106.4
C2—C1—C8	119.1 (2)	C1—C8—H8	106.4
C12—C1—C8	112.4 (2)	C7—C8—H8	106.4
C2—C1—C3	59.50 (16)	C10—C9—C8	124.6 (2)
C12—C1—C3	122.2 (2)	C10—C9—H9	117.7
C8—C1—C3	117.4 (2)	C8—C9—H9	117.7
C3—C2—C1	60.98 (16)	C9—C10—C16	119.1 (2)
C3—C2—Cl2	121.47 (19)	C9—C10—C11	122.9 (2)
C1—C2—Cl2	121.68 (18)	C16—C10—C11	118.0 (2)
C3—C2—Cl1	119.12 (19)	C10—C11—C12	112.2 (2)
C1—C2—Cl1	119.24 (18)	C10—C11—H11A	109.2
Cl2—C2—Cl1	108.12 (14)	C12—C11—H11A	109.2
C13—C3—C4	113.4 (2)	C10—C11—H11B	109.2
C13—C3—C2	119.0 (2)	C12—C11—H11B	109.2
C4—C3—C2	118.0 (2)	H11A—C11—H11B	107.9
C13—C3—C1	120.5 (2)	C1—C12—C11	108.8 (2)
C4—C3—C1	116.3 (2)	C1—C12—H12A	109.9
C2—C3—C1	59.51 (16)	C11—C12—H12A	109.9
C3—C4—C5	111.7 (2)	C1—C12—H12B	109.9
C3—C4—H4A	109.3	C11—C12—H12B	109.9
C5—C4—H4A	109.3	H12A—C12—H12B	108.3
C3—C4—H4B	109.3	C3—C13—H13A	109.5
C5—C4—H4B	109.3	C3—C13—H13B	109.5
H4A—C4—H4B	107.9	H13A—C13—H13B	109.5
C4—C5—C6	114.7 (2)	C3—C13—H13C	109.5
C4—C5—H5A	108.6	H13A—C13—H13C	109.5
C6—C5—H5A	108.6	H13B—C13—H13C	109.5
C4—C5—H5B	108.6	C7—C14—H14A	109.5
C6—C5—H5B	108.6	C7—C14—H14B	109.5
H5A—C5—H5B	107.6	H14A—C14—H14B	109.5
C7—C6—C5	118.3 (2)	C7—C14—H14C	109.5
C7—C6—H6A	107.7	H14A—C14—H14C	109.5
C5—C6—H6A	107.7	H14B—C14—H14C	109.5
C7—C6—H6B	107.7	C7—C15—H15A	109.5
C5—C6—H6B	107.7	C7—C15—H15B	109.5
H6A—C6—H6B	107.1	H15A—C15—H15B	109.5
C14—C7—C6	111.1 (2)	C7—C15—H15C	109.5
C14—C7—C15	107.7 (3)	H15A—C15—H15C	109.5
C6—C7—C15	106.7 (2)	H15B—C15—H15C	109.5
C14—C7—C8	107.5 (2)	C10—C16—Br1	112.15 (18)
C6—C7—C8	112.1 (2)	C10—C16—H16A	109.2
C15—C7—C8	111.7 (2)	Br1—C16—H16A	109.2
C9—C8—C1	109.8 (2)	C10—C16—H16B	109.2
C9—C8—C7	112.2 (2)	Br1—C16—H16B	109.2
C1—C8—C7	115.1 (2)	H16A—C16—H16B	107.9
